# CFD assisted investigation of mechanical juice extraction from cassava leaves and characterization of the products

**DOI:** 10.1002/fsn3.1517

**Published:** 2020-03-13

**Authors:** Sajid Latif, Sebastian Romuli, Ziba Barati, Joachim Müller

**Affiliations:** ^1^ Institute of Agricultural Engineering (440e) Tropics and Subtropics Group University of Hohenheim Stuttgart Germany

**Keywords:** amino acids, cassava leaves, characterization, computational fluid dynamics, mechanical pressing

## Abstract

Cassava is grown because of its starchy roots, but the leaves being rich in protein are mostly underutilized. For protein recovery, mechanical juice extraction from cassava leaves and the extraction process was evaluated using computational fluid dynamics (CFD) simulation. The influence of input variables such as nozzle diameter and rotational speed of the screw was investigated in relation to process efficiency. The highest green juice extraction yield (81.0%) from cassava leaves and dry matter of press cake (61.3%) were achieved by using 4 mm nozzle diameter and 18 rpm screw speed. The protein content of the cassava leaves, press cake, juice sediment, and juice supernatant was found to be 31.5%, 27.7%, 26.2%, and 12.4%, respectively. The crude protein, cellulose, hemicellulose, lignin, and total phenolic content mainly accumulated in the press cake. The screw pressing concentrated the amino acids in the press cake and the juice sediment.

## Graphical abstract

Simulation of flow behavior and heat transfer of cassava leaf puree was conducted. Best nozzle size (4 mm) and screw speed (18 rpm) have been identified. The crude protein, TPC, cellulose, hemicellulose, and lignin content accumulated in the press cake. Highest protein content was found in press cake followed by the juice sediment and juice supernatant. The amino acids were not negatively affected and were concentrated in press cake and juice sediment.

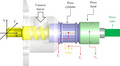

## 1 INTRODUCTION

Cassava (*Manihot esculenta* Crantz) ranks as one of the most important crops, grown in 105 countries mainly for its starchy roots. It is a staple food for nearly one billion people and is considered as one of the most important source of diet in tropical countries (Latif & Müller, 2015). In addition to the roots, a huge economic potential lies in cassava leaves which contain 17%–38% protein on dry weight basis. Cassava leaves as an additional protein source also form a major part of diet in some regions but in many countries they are not consumed at all due to low digestibility, antinutrients, toxicity, and taste (Urribarrí, Chacón, González, & Ferrer, 2009). Cassava leaves, which are mostly left in the field, could be a sustainable source of protein, minerals, and vitamins and hence could contribute to food and nutrition security.

In a green bio‐refinery approach, protein can be extracted from the leaves with possible utilization in food, animal feed, packaging material, and bulk chemicals (after hydrolysis) while the remaining fiber can be used as a feedstock for bioethanol or as roughage for ruminants (Swieca, Seczy, Gawlik‐Dziki, & Dziki, 2014; Zhang, Sanders, & Bruins, 2014). After suitable processing, cassava leaf protein can be recovered while removing the antinutritional factors. Various methods including aqueous extraction with subsequent ultrafiltration (Dale, Allen, Laser, & Lynd, 2009), enzymatic extraction (Vergara‐Barberán, Lerma‐García, Herrero‐Martínez, & Simó‐Alfonso, 2015), enzymatic extraction coupled with ammonia treatment (Urribarrí et al., 2009), microwave‐assisted extraction (Thirugnanasambandham, Sivakumar, & Maran, 2015), and mechanical extraction (Colas, Doumeng, Pontalier, & Rigal, 2013) have been developed for leaf protein recovery.

Mechanical protein extraction from leaves using screw presses is considered as a suitable approach for rural areas due to its affordability and simplicity (Telek, 1983). On the other hand, mechanical fractionation of green raw material is essential process in a green bio‐refinery plant (Mahmoud, Arlabosse, & Fernandez, 2011). In general, the freshly harvested leaves are chopped or ground, squeezed by mechanical pressing followed by heating or iso‐electric precipitation of the juice to recover the protein (Carlsson & Hanczakowski, 1989). The resulting protein is called leaf protein concentrate (LPC). In order to efficiently extract protein from the leaves, the plant cells must be disrupted through mechanical pressing (Telek, 1983). Therefore, the efficiency of the screw press is one of the major factors, which limit the economic performance of mechanical protein extraction.

Concerning mechanical extraction of cassava leaf juice, rotational speed of the screw and nozzle diameter is the main influencing factors in terms of process efficiency (Ferrari, Leonel, & Mischan, 2014). The geometrical characteristics of a screw press such as screw pitch and thread depth also have an impact on the extraction performance (Ficarella, Milanese, & Laforgia, 2006). During pressing, related parameters such as throughput, pressure, and temperature should be taken into account as they influence friction and shearing processes of the material in an expeller (Karaj & Müller, 2011).

In order to conduct the numerical evaluation and to investigate the flow behavior of the material, cassava leaves were considered as non‐Newtonian fluid (Favier, 2014). Calculations are based on Navier–Stokes equations that have been widely used for extrusion processes (Gopalakrishna, Jaluria, & Karwe, 1992; Kwag, Kim, Lee and Lyu, 2001), cotton seed cake (He & Zhou, 2010), wheat dough (Dhanasekharan & Kokini, 2003), and liquid food (Kechichian, Crivellari, Gut, & Tadini, 2012). The computational approach seems to be promising for designing efficient screw presses for cassava leaves.

The objective of this study was to evaluate the effect of nozzle size and screw speed of a screw press in order to maximize cassava juice extraction from cassava leaves. CFD simulation of flow behavior and heat transfer of cassava leaf puree as non‐Newtonian fluid were established to reduce experimental work and to allow transfer of results to changing frame conditions. The physicochemical characteristics and the amino acid profile of the products obtained at the best pressing conditions were analyzed and compared with that of untreated cassava leaves.

## 2 MATERIAL AND METHODS

### 2.1 Characterization of cassava leaves and its products after screw pressing

Frozen cassava leaves were acquired from an Africa‐shop in Stuttgart, Germany. The cassava leaves were stored at −18°C and thawed for 24 hr at room temperature before being used for the experiments.

Total phenolic content (TPC) was measured according to Folin–Ciocalteu method of Singleton, Orthofer, and Lamuela‐Raventós (1999) with slight modification. As a reference standard for TPC, gallic acid solution was used. TPC was expressed as gallic acid equivalent per gram of dry weight (mg GAE/g). The color was determined in terms of CIELAB values using colorimeter (CR‐400, Konica Minolta). Cellulose, hemicellulose, and lignin content were determined according to Van Soest, Robertson, and Lewis (1991) method. Crude fiber was determined according to buffered acid‐detergent procedures (AOAC, 1990). Crude protein content was calculated as N × 6.25 with Kjeldahl method (AOAC, 1995). Essential and nonessential amino acids were analyzed according to Commission Regulation (EC) No 152/2009, Annex III, Method F&G by ion exchange chromatography using C_9_H_6_O_4_ (Ninhydrin) postcolumn derivatization.

### 2.2 Mechanical screw pressing of cassava leaf

A mechanical screw press CA59G (IBG Monforts Oekotec GmbH & Co. KG, Mönchengladbach, Germany) was used for extracting green juice from 1 kg cassava leaves per trial. The screw had a diameter of 33.9 mm, a thread depth of 5.6 mm, and a pitch of 17.9 mm. The press cylinder had a hole diameter of 1 mm, and there were 120 holes on an area of 47.12 cm^2^.

Rotational speed of the electric motor, *ω_m_* (rpm) and screw speed, *ω_s_* (rpm) were measured using a digital tachometer DT‐2234 (ZEITECH, Pfaffenhofen, Germany). The relation between *ω_s_* and *ω_m_* was as follows:

(1) $$\omega_{s}=0.092\cdot \omega_{m}$$

Nozzle diameter, *Ø_N_* (mm) and screw speed, *ω_s_* were considered as the main operating parameters. Nozzle diameter was set at 4, 5, and 6 mm, while screw speed at 18, 28, and 40 rpm as the low, middle, and high level, respectively.

Throughput, *TP* (kg/hr) as the mass flow rate of cassava leaves for each trial was estimated as follows:

(2) $$TP=\frac{m_{\mathrm{cl}}}{t}$$

where *m_cl_* (kg) is the initial sample mass of cassava leaves, and *t* (h) is the duration of the extraction.

Green juice extraction yield, *Y* (%) was estimated based on mass of cassava leaves and recovered green juice as follows:

(3) $$Y=\frac{m_{\mathrm{gj}}}{m_{\mathrm{cl}}}\cdot 100$$

where *m_gj_* (kg) is the mass of green juice.

**FIGURE 1:**
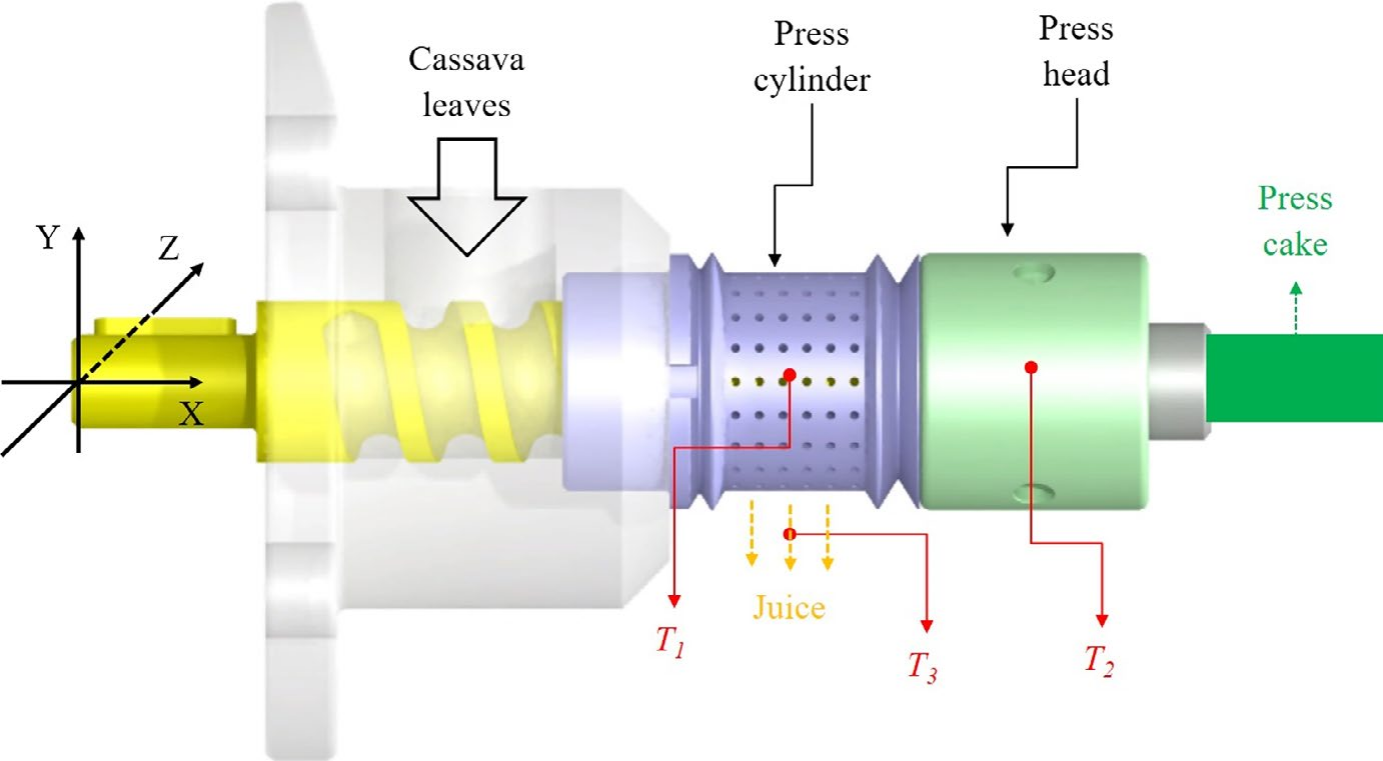
Mechanical screw press CA59G and temperature measurement of press cylinder (*T_1_*), press head (*T_2_*), and green juice (*T_3_*)

Temperature of press cylinder, *T_1_,* press head, *T_2_* and green juice, *T_3_* (^o^C) were measured using type K (NiCr‐Ni) thermocouples (GHM Messtechnik GmbH, Regenstauf, Germany) as shown in Figure 1. The measurement was recorded using a digital data logger 34970A (Agilent Technologies) at an interval of 10 s.

### 2.3 Flow simulation of cassava leaf puree as non‐Newtonian fluid

Cassava leaf puree was considered as non‐Newtonian fluid, and the flow was assumed to be nonisothermal and in a steady‐state condition in the press head. As no information about rheological properties of cassava leaf puree was found, data of mint puree were adopted as published by Rudra, Sarkar, Shivhare, and Basu (2008). Thermophysical properties such as thermal conductivity, *k* (W/m·K) and specific heat, *C_p_* (J/kg·K) were adopted from spinach (Bergeron Quirion, Villeneuve, Leblanc, & Delaquis, 2012).

Flow simulation was carried out using CFD software Solidworks 2014 (Dassault Systèmes, Vèlizy‐Villacoublay, France). Simulation of the cassava leaf pressing process was carried out at constant speed of 18 rpm, and 4 mm nozzle diameter, whereas *TP* was taken from the experiments. Outer surface of press head was taken as the upper boundary of computational domain, and its initial temperature was assumed to be equal to the initial temperature of the inner surface. The pressing area in the press head was determined as the computational domain. Screw and press head surface were assumed to be adiabatic. Thermal boundary and iteration steps of pressure in the press head were done as described by He and Zhou (2010). Atmospheric pressure (1.013 bar) was assumed at the outlet of the nozzle.

Primary flow direction was in the *x*‐direction (See Figure 1). The details of the flow schematic diagram in press head channel were in accordance with a scientific work conducted by Kwag et al. (2001). The computational evaluation was conducted based on conservation laws for mass, angular momentum and energy, which are formulated in Navier–Stokes equations (Dhanasekharan & Kokini, 2003; Kwag et al., 2001):

(4) $$\rho \cdot \frac{\partial u_{i}}{\partial x_{i}}=0$$

(5) $$\frac{\partial \left(\rho \cdot u_{i}\right)}{\partial t}+\frac{\partial \left(\rho \cdot u_{i}\cdot u_{j}\right)}{\partial x_{i}}+\frac{\partial p}{\partial x_{i}}=\frac{\partial \tau_{\mathrm{ij}}}{\partial x_{j}}+S_{i}$$

where *x_i_* and *x_j_* (m) refer to Cartesian coordinates of pixel position of row *i* and column *j* (*i* = 1, 2, 3; *j* = 1, 2, 3), *p* (Pa) is pressure, *τ_ij_* is viscous shear stress tensor, and *u_i_* (m/s) is velocity in *x_i_* direction. *S_i_* is the sum of mass‐distributed external force per unit mass due to porous media resistance, *S_i_^porous^*, rotation of system coordinate, *S_i_^rotation^*, and buoyancy, *S_i_^gravity^* which is estimated as:

(6) $$S_{i}^{\mathrm{gravity}}=-\rho \cdot g_{i}$$

where *g_i_* is gravitational acceleration component along the *i*‐th coordinate direction.

Estimation of heat transfer is adapted from enthalpy conservation equation in single screw extruder as follows:

$$\frac{\partial \left(\rho \cdot H\right)}{\partial t}+\frac{\partial \left(\rho \cdot u_{i}\cdot H\right)}{\partial x_{i}}=\frac{\partial \left(u_{i}\cdot \tau_{\mathrm{ij}}+q_{i}\right)}{\partial x_{i}}+\frac{\partial p}{\partial t}+\rho \cdot \varepsilon +S_{i}\cdot u_{i}+Q_{H},$$

where

(7) $$H=h+\frac{u^{2}}{2}$$

where *h* is enthalpy (J), *Q_H_* is volumetric heat capacity (J/m^3^·K), *ε* is turbulent dissipation, and *q_i_* is diffusive heat flux (J/s) calculated from thermal conductivity and temperature as:

(8) $$q_{i}=-k\cdot \frac{\partial T}{\partial x_{i}}$$

As for non‐Newtonian fluid, *τ_ij_* is estimated in Equation 9:

(9) $$\tau_{\mathrm{ij}}=\mu \left(\gamma \right)\cdot \left(\frac{\partial u_{i}}{\partial x_{j}}+\frac{\partial u_{j}}{\partial x_{i}}\right)$$

Shear rate, *γ* (1/s) has the following form:

(10) $$\gamma =\sqrt{d_{\mathrm{ij}}^{2}-d_{\mathrm{ii}}\cdot d_{\mathrm{jj}},}\;\text{where}\;d_{\mathrm{ij}}\;=\;\frac{\partial u_{i}}{\partial x_{j}}+\frac{\partial u_{j}}{\partial x_{i}}$$

*μ*(*γ*) is viscosity function over shear rate specified according to Herschel–Bulkley model:

(11) $$\mu \left(\gamma \right)=K\cdot \gamma^{n-1}+\frac{\tau_{0}}{\gamma }$$

where *K* is the consistency coefficient, *n* power law index, and *τ_0_* yield stress of the fluid (Pa).

### 2.4 Statistical analysis

Analyses were made in three replicates, and the average values were reported in this study. As for statistical analysis, coefficient of correlation, *R* ^2^ and root mean square error, *RMSE* were determined to estimate the accuracy of the mathematical model. The data were plotted by using Origin Pro 9 (OriginLab Co.). An analysis of variance (ANOVA) was performed with the statistic software SPSS (version 24, SPSS Inc., IBM) to test for differences among samples and the least significant difference (LSD) statistical analysis to establish which samples were statistically different. A level of significance of 0.05 was used on all statistical analysis.

## 3 RESULTS AND DISCUSSION

### 3.1 CFD flow simulation

**FIGURE 2:**
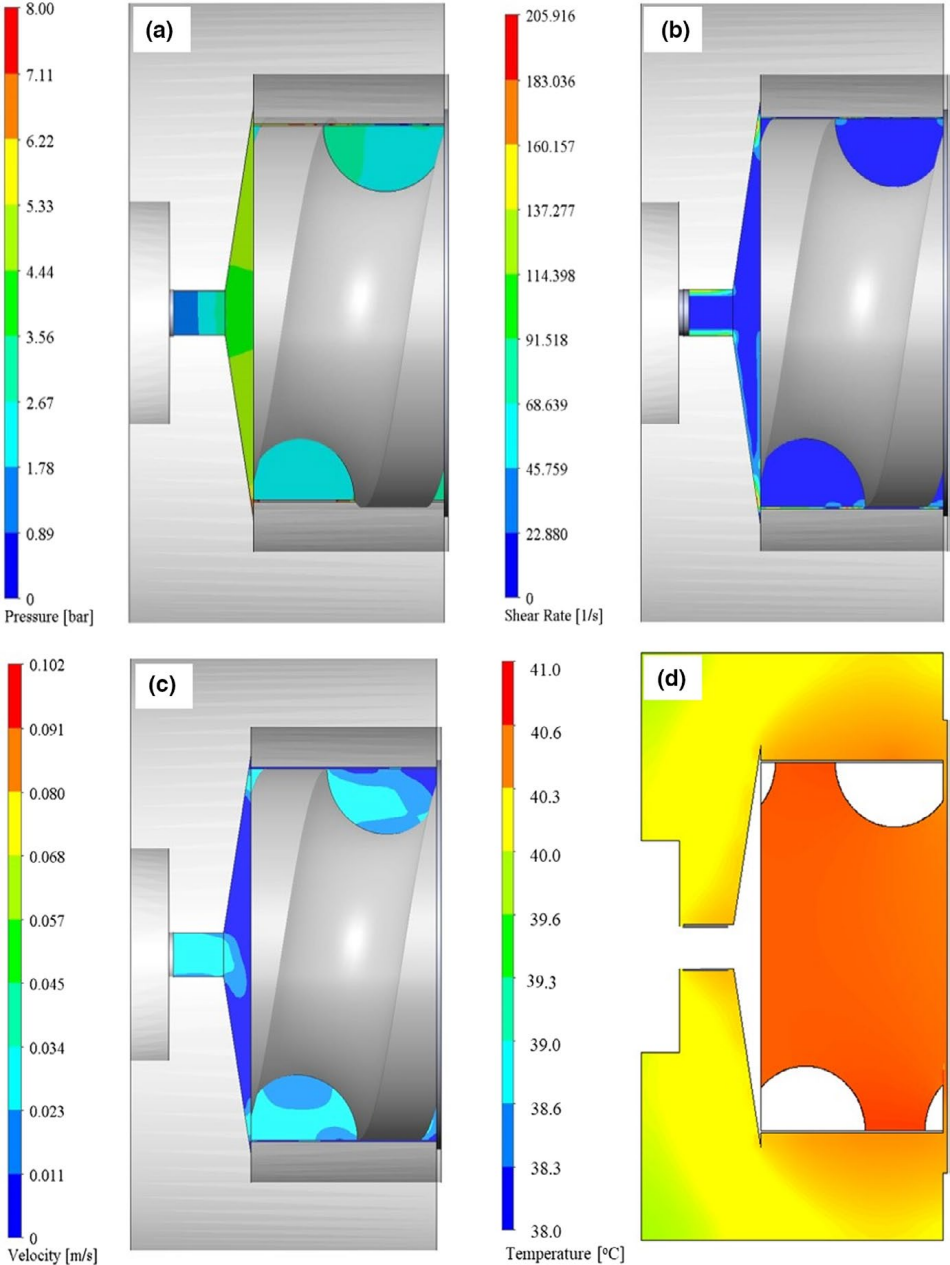
Computational fluid dynamics (CFD) simulation of pressure (a), shear rate (b), magnitude velocity (c), and temperature distribution in press head (d) (*Ø_N_* = 4 mm, *ω_s_* = 18 rpm)

Pressure and shear rate in the press head are demonstrated in Figure 2. The highest pressure (5.23 bar) was achieved at the inner part of nozzle due to the fluid accumulation pushed by the screw press on the inner part of the nozzle (Figure 2a). However, the pressure was lower than the mechanical extraction of J. curcas seeds (Karaj & Müller, 2011), due to the soft texture of cassava leaves. A drastic decrease in pressure is observed from the inner to outer part of the nozzle.

The shear rate is substantially affected by rotational speed (Figure 2b), and similar patterns were also obtained for cereals as reported by Ficarella et al. (2006). Overall, a low shear rate was observed which may be attributed to a low rotational speed of the screw and texture characteristics of cassava leaf puree. Shear rate can be increased by reducing depth of thread leading to a less volume of the screw channel, as suggested by Chiruvella, Jaluria, and Karwe (1996).

Magnitude velocity in the press head is presented in Figure 2c. The dark gray area on the top and bottom side of the screw press (Figure 2c) represents the external thread of press cylinder, which was inserted to the threaded inner part of the press head. The puree is flowing parallel to the movement of the screw. The predicted velocity of press cake coming out of the nozzle was 0.027 m/s, which was close to the measured velocity, namely 0.021 m/s. The dark blue area, which indicated low velocity below 0.011 m/s, was mostly concentrated at the narrow area between the planar end surface of the screw press and the nozzle surface inside the press head. This was the area where the leaves were pressed under maximum pressure before entering the nozzle.

Figure 2d demonstrates the numerical heat transfer from the pressing chamber to the surface of the press head. The simulated press head temperature (40.6°C) was found close to the average experimental temperature (44.7°C) at steady‐state condition.

The generated pressure and press head temperature from screw pressing of cassava leaf were much lower than those of an extrusion system of rice starch with high moisture content (> 55%), as reported by Akdogan (1996). Moreover, flow modeling seemed to be a suitable method for improving the geometry of screw press in terms of pitch diameter, thread depth, screw diameter, and helix angle.

### 3.2 Measured temporal and spatial temperature distribution

**FIGURE 3:**
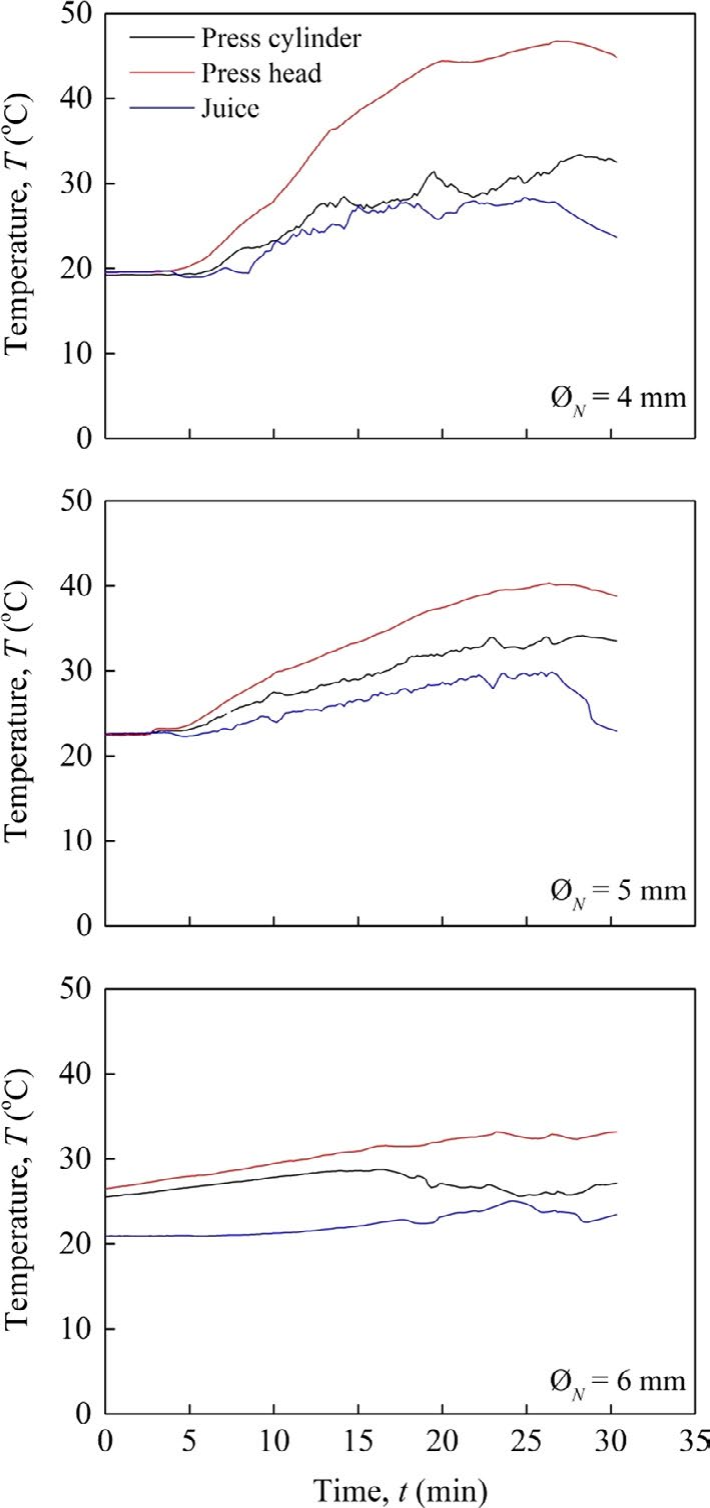
Temperature, *T* of extraction process at 18 rpm *ω_s_* for nozzle diameters, *Ø_N_* of 4 mm (top), 5 mm (middle), and 6 mm (bottom)

Figure 3 shows the effect of different nozzle diameters (4, 5, and 6 mm) on the temperature of the press cylinder, press head, and green juice. At steady‐state conditions for the smallest nozzle diameter (4 mm), the highest temperatures of 33.3 and 45.4°C were observed for press cylinder and press head, respectively (Figure 3). It took around 10–15 min to reach this temperature from the initial temperature (20°C) of the press cylinder and press head with a throughput of 1.0 − 1.1 kg/hr. This may be attributed to the higher pressure and friction in the cylinder due to the small opening. A similar trend was observed for the temperatures of press cylinder and press head while changing the nozzle size from 5 to 6 mm. However, no significant difference was found in green juice temperature at different nozzle diameters, which may be due to a rapid cooling of the juice by the lower ambient temperature while flowing out from the press cylinder.

The heat at the press head was generated by friction force between the material and inner wall of the press head. However, the overall temperature was below 50°C due to the soft texture and high water content of cassava leaves, which allowed moderate friction against the inner wall of the cylinder. The average experimental temperature at steady‐state condition (44.7°C) was found close to the simulated press head temperature. Those temperatures generated during cassava leaves pressing were lower than heating condition during thermally assisted mechanical dewatering of alfalfa (Mahmoud et al., 2011) and spinach (Arlabosse, Blanc, Kerfaï, & Fernandez, 2011).

### 3.3 Performance of mechanical extraction of cassava leaves

**FIGURE 4:**
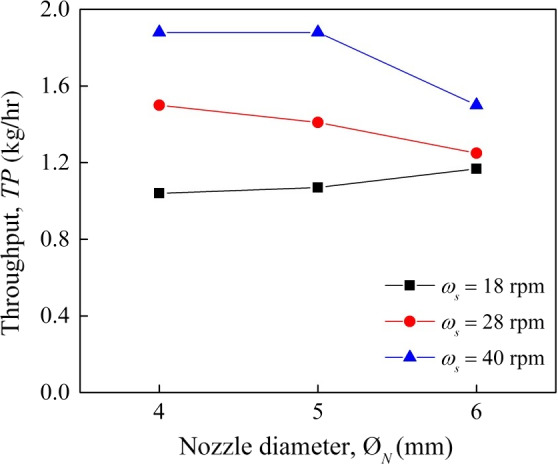
Throughput *S* (kg/hr) at different nozzle diameter *Ø_N_* (mm) with respect to screw speed *ω_s_* (rpm)

In this study, the influence of screw press speed and nozzle diameter on throughput shows that higher speed led to higher throughput (Figure 4). At screw speed of 18 rpm, throughput increased slightly from 1.0 to 1.1 kg/hr as nozzle diameter was enlarged from 4 to 5 mm. The highest throughput (1.9 kg/hr) was achieved at high screw speed and when nozzle diameter was set to 4 mm.

At screw speed of 28 and 40 rpm, throughput decreased by increasing the nozzle diameter, which may be due to a low pressure in the pressing chamber and less ability to densify the press cake coming out of the nozzle. Inside the expeller, cassava leaves turned into adhesive leaf paste due to high moisture content of the leaves. Higher screw speed with larger nozzle diameter lead to lower pressure and friction resulting in a higher amount of the paste attached to the screw press and kept rotating with it. On the other hand, this transport phenomenon also resulted in higher velocity of the paste that had less contact with the inner wall of the expeller and the outer wall of the screw press. Therefore, the velocity of the leaves through the expeller was obviously higher under higher screw speed, but lower in terms of throughput.

**FIGURE 5:**
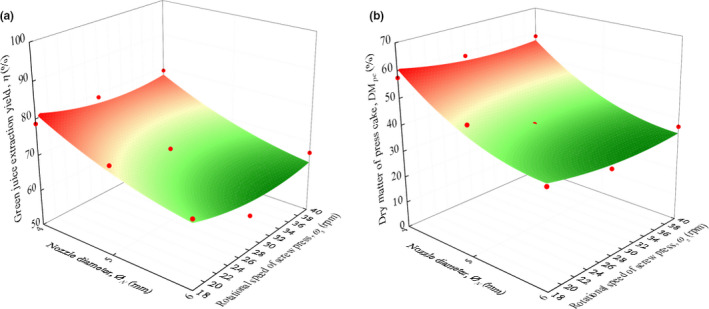
Effect of nozzle diameter, *Ø_N_* and the screw press speed, *ω_s_* on green juice extraction yield (a), *Y* (*R* ^2^ = 0.845) and dry matter content of press cake (b), *DM_pc_* (*R* ^2^ = 0.934)

Figure 5 demonstrates the effect of nozzle diameter and screw speed on green juice extraction yield and dry matter of the press cake. The correlations for yield and dry matter content for nozzle diameter and screw speed are given in Equations 12 and 13

(12) $$Y=173.74-26.37\cdot {\text{Ø}}_{N}-1.37\cdot \omega_{s}+1.95\cdot {\text{Ø}}_{N}^{2}+0.02\cdot \omega_{s}^{2}$$

(13) $$DM_{\mathrm{pc}}=248.94-63.91\cdot {\text{Ø}}_{N}-1.25\cdot \omega_{s}+5.25\cdot {\text{Ø}}_{N}^{2}+0.02\cdot \omega_{s}^{2}$$

The adequacy of the models for green juice extraction yield and dry matter of press cake is indicated by a coefficient of correlation *R^2^* = 0.845 and 0.934, and *RMSE* = 3.854 and 3.927, respectively.

Low water content was observed in the press cake by using low rotational speed and small nozzle diameter. Nozzle diameter smaller than 4 mm was not possible as there was a tendency of clogging the nozzle, with the consequence of squeezing out the press cake through the press cylinder. Highest extraction yield (81.0%) and dry matter content (61.3%) of the press cake was achieved by using 4 mm nozzle diameter and 18 rpm screw speed. This extraction yield of green juice from cassava leaves was well in line with that of spinach (81%) but higher than that of alfalfa (62.5%), both obtained by batch process as reported by Arlabosse et al. (2011). Dry matter content of cassava press cake (61.3%) was slightly lower than that of alfalfa press cake (66%) as reported by Mahmoud et al. (2011).

### 3.4 Characterization of cassava leaves and its products after pressing

The physicochemical properties of cassava leaves and its products, that is, press cake, juice sediment, and juice supernatant obtained at best pressing conditions (at the screw speed of 18 rpm and the nozzle diameter of 4 mm) are presented in Table 1. The result of mass balance showed that the juice supernatant constitutes the highest share followed by the press cake and the juice sediment. Due to high amount of minerals in cassava leaves (Latif & Müller, 2015), it has higher ash content in comparison with other green leafy vegetables (Goyeneche et al., 2015). The juice supernatant had the highest ash content in comparison with that of juice sediment, press cake, and cassava leaves. These results are similar to the findings of Tenorio, Gieteling, De Jong, Boom, and Van Der Goot (2016).

**Table 1:** Physicochemical properties of cassava leaves, press cake, and juices (sediment, supernatant)

	Mass balance % FM	Dry matter % FM	Ash % DM	TPC^a^ mg GAE/g	Cellulose % DM	Hemicellulose % DM	Lignin % DM	Crude fiber % DM	Crude protein % DM	L*	a*	b*
Cassava leaves	100^d^	13.0 ± 0.3^c^	5.3 ± 0.1^b^	14.2 ± 0.8^d^	13.9 ± 0.6^b^	12.6 ± 0.5^a^	7.4 ± 0.2^b^	13.6 ± 0.9^b^	31.5 ± 0.2^d^	34.6 ± 0.0^b^	−3.1 ± 0.0^a^	6.3 ± 0.0^b^
Press cake	18.5 ± 1.9^b^	58.2 ± 0.5^d^	3.6 ± 0.1^a^	11.4 ± 0.9^c^	14.9 ± 0.9^b^	19.0 ± 0.4^b^	9.7 ± 0.2^c^	17.5 ± 0.9^c^	27.7 ± 0.3^c^	42.0 ± 0.0^d^	−2.4 ± 0.0^c^	8.7 ± 0.02^c^
Juice sediment	12.7 ± 1.0^a^	10.9 ± 0.0^b^	6.3 ± 0.1^c^	0.4 ± 0.04^b^	7.7 ± 0.6^a^	10.8 ± 0.8^a^	4.8 ± 1.1^a^	3.6 ± 0.3^a^	26.2 ± 0.1^b^	37.1 ± 0.01^c^	−2.7 ± 0.0^b^	15.5 ± 0.02^d^
Juice supernatant	64.4 ± 3.0^c^	2.4 ± 0.6^a^	15.6 ± 0.2^d^	0.1 ± 0.01^a^	_	_	_	_	12.4 ± 0.5^a^	29.6 ± 0.01^a^	−1.2 ± 0.02^d^	4.5 ± 0.02^a^

*Note*: Mean values in columns followed by the same letter are not significantly different (*p* > .05).

^a^ Total phenolic contents (TPC): mg Gallic acid equivalent/g.

The TPC in cassava leaves was lower than that of white variety yacon leaves, namely 46.5 – 59.2 mg GAE/g (Khajehei et al., 2017) but higher than that of radish leaves (6.9 mg GAE/g) (Goyeneche et al., 2015). The amount of TPC significantly (*p* < .05) decreased in the press cake compared with the cassava leaves (Table 1) since some amount of TPC distribute in the juice supernatant and sediment. Moreover, the amount of TPC decreased during processing and storage (Ellong, Billard, Adenet, & Rochefort, 2015). Very low content of TPC in the juice supernatant and juice sediment suggests that TPC of cassava leaves may belong to insoluble‐bound phenolics.

It was found that the cellulose and lignin contents were significantly (*p* < .05) reduced in juice sediment as compared to cassava leaves and press cake. The highest hemicellulose content was found in the press cake followed by cassava leaves and juice sediment. There were no significant (*p* > .05) differences in cellulose contents of cassava leaves and press cake. A reasonable amount of protein was present in press cake (27.7%), juice sediment (26.2%), and juice supernatant (12.4%) (Table 1). Tenorio et al. (2016) also reported that after screw pressing of green leaves, fibrous pulp (press cake) contained the highest amount of protein (31.1%) followed by juice sediment (29.2%) and juice supernatant (13.6%).

Color plays a significant role in indicating the quality of food materials due to considerable influence on visual appearance, processing, and acceptability. Furthermore, the nutrient content of the food can determine the color. For example, the significant changes in CIELAB parameters a* and b* (browning) of Plantago lanceolata L. leaves during long‐term storage were reported as a result of conversion of chlorophyll to pheophytin (Gonda et al., 2012). The results of color measurements in Table 1 showed that cassava leaves had significantly (*p* < .05) high intensive green color followed by juice sediment, press cake, and juice supernatant. The b* value and L* value ranged from 4.5 to 15.5 (yellowish) and from 29.6 to 42 (lightness), respectively. Based on other studies, different food processing would affect the color values (Kupongsak & Manomaiwajee, 2016). In this study, the screw pressing of cassava leaves significantly (*p* < .05) influenced the color values. This change in color could be related to the change of nutrients content of the samples such as TPC, amino acids, chlorophyll, or due to the presence of membrane proteins (Kupongsak & Manomaiwajee, 2016; Tenorio, Boom, & Goot, 2017).

**FIGURE 6:**
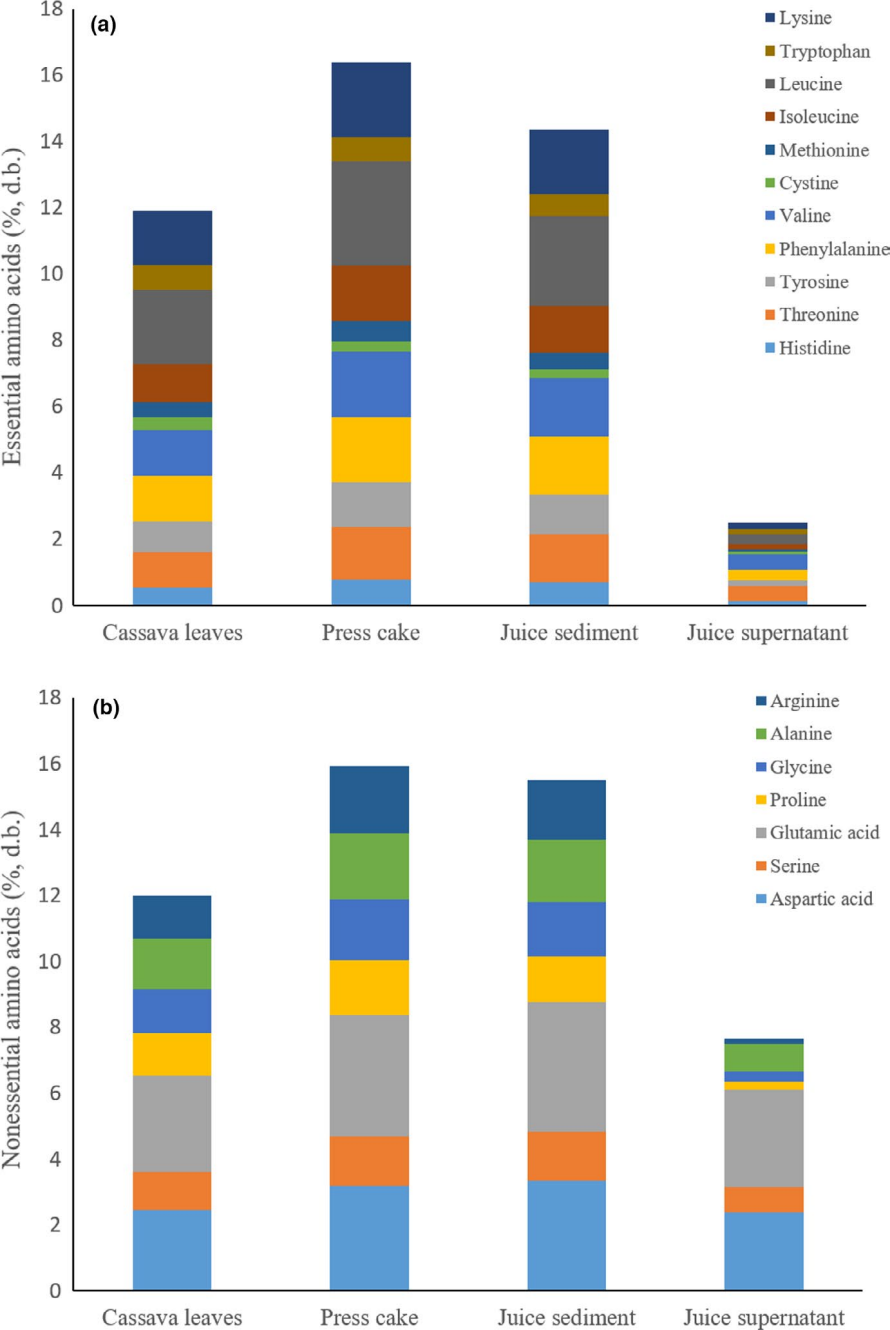
The essential (a) and nonessential amino acids (b) of cassava leaves, press cake, and juices (supernatant, sediment)

Figure 6 shows the essential amino acids (EAAs) and nonessential amino acids (NAAs) of cassava leaves, press cake, juice sediment, and juice supernatant. The press cake had the highest amount of amino acids followed by juice sediment, cassava leaves, and juice supernatant. The concentrations of both EAAs and NAAs were increased in the press cake and the juice sediment but decreased in the juice supernatant. Glutamic acid was dominant while methionine and cystine were the limiting amino acids in cassava leaves and the products obtained after pressing. Generally, the amino acid profiles of native plant proteins are similar to small differences due to age of the plant. Nevertheless, the extraction method influenced the composition of the extracted protein, especially heat may cause cross‐linkages between amino acids and lead to amino acid losses (Chiesa & Gnansounou, 2011). In alfalfa protein, racemization of amino acids was reported by Schwass and Finley (1984). Contrarily, the heat produced in our process did not negatively affect the EAAs and NAAs.

## 4 CONCLUSIONS

Computational fluid dynamics flow simulation of cassava leaf pressing, considering the leaf puree as non‐Newtonian fluid seems to be a viable option. It exemplifies that high screw speed leads to high velocity of material, but not always refers to high throughput since transport phenomena also depend on the nozzle diameter. The numerical approach in this study also showed good potential in predicting pressure and flow trajectories during extraction in laminar condition. CFD simulation allowed us to estimate parameters that cannot be measured during experiments, and as a decision‐support tool to further develop the geometry of the screw press, press cylinder, and nozzle. Yield of green juice cannot be simulated, since the rheological properties of the green juice are not known. It is recommended to investigate thermophysical and rheological properties of cassava leaf puree to increase the accuracy of simulation results. It was found that the lower levels of the screw speed (18 rpm) and the nozzle diameter (4 mm) lead to a higher green juice extraction yield and dry matter content of press cake. In order to avoid any blockage during cassava leaf pressing, it is recommended to use a nozzle diameter not smaller than 4 mm. The screw pressing did not negatively affect both EAAs and NAAs and concentrated them in press cake and juice sediment. The crude protein, TPC, cellulose, hemicellulose, and lignin content mainly accumulated in the press cake, which can be considered for animal feeding. However, the juice sediment and the juice supernatant can be further processed for human food.

## CONFLICT OF INTEREST

The authors declare no conflict of interest.

## ETHICAL APPROVAL

This study does not involve any human or animal testing.
